# Synthetic lethal connectivity and graph transformer improve synthetic lethality prediction

**DOI:** 10.1093/bib/bbae425

**Published:** 2024-08-30

**Authors:** Kunjie Fan, Birkan Gökbağ, Shan Tang, Shangjia Li, Yirui Huang, Lingling Wang, Lijun Cheng, Lang Li

**Affiliations:** Department of Biomedical Informatics, College of Medicine, The Ohio State University, 1800 Cannon Drive, Columbus, OH 43210, United States; Department of Biomedical Informatics, College of Medicine, The Ohio State University, 1800 Cannon Drive, Columbus, OH 43210, United States; Department of Biomedical Informatics, College of Pharmacy, The Ohio State University, 500 W. 12 ave, Columbus, OH 43210, United States; Department of Biomedical Informatics, College of Medicine, The Ohio State University, 1800 Cannon Drive, Columbus, OH 43210, United States; Department of Biomedical Informatics, College of Pharmacy, The Ohio State University, 500 W. 12 ave, Columbus, OH 43210, United States; Department of Biomedical Informatics, College of Medicine, The Ohio State University, 1800 Cannon Drive, Columbus, OH 43210, United States; Department of Biomedical Informatics, College of Medicine, The Ohio State University, 1800 Cannon Drive, Columbus, OH 43210, United States; Department of Biomedical Informatics, College of Medicine, The Ohio State University, 1800 Cannon Drive, Columbus, OH 43210, United States; Department of Biomedical Informatics, College of Pharmacy, The Ohio State University, 500 W. 12 ave, Columbus, OH 43210, United States

**Keywords:** CRISPR-Cas9 double knockout, deep learning, synthetic lethality

## Abstract

Synthetic lethality (SL) has shown great promise for the discovery of novel targets in cancer. CRISPR double-knockout (CDKO) technologies can only screen several hundred genes and their combinations, but not genome-wide. Therefore, good SL prediction models are highly needed for genes and gene pairs selection in CDKO experiments. However, lack of scalable SL properties prevents generalizability of SL interactions to out-of-sample data, thereby hindering modeling efforts. In this paper, we recognize that SL connectivity is a scalable and generalizable SL property. We develop a novel two-step multilayer encoder for individual sample-specific SL prediction model (MLEC-iSL), which predicts SL connectivity first and SL interactions subsequently. MLEC-iSL has three encoders, namely, gene, graph, and transformer encoders. MLEC-iSL achieves high SL prediction performance in K562 (AUPR, 0.73; AUC, 0.72) and Jurkat (AUPR, 0.73; AUC, 0.71) cells, while no existing methods exceed 0.62 AUPR and AUC. The prediction performance of MLEC-iSL is validated in a CDKO experiment in 22Rv1 cells, yielding a 46.8% SL rate among 987 selected gene pairs. The screen also reveals SL dependency between apoptosis and mitosis cell death pathways.

## Graphical Abstract

**Graphical Abstract:**
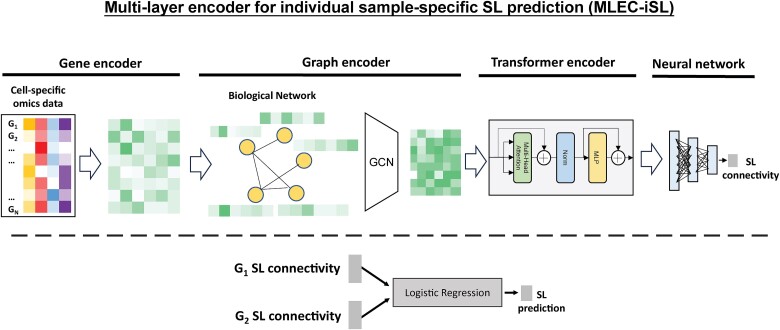

## Introduction

Synthetic lethality (SL) describes a functional relationship between two genes in a cell associated with the cell’s viability; when only one of the genes is lost, the cell remains viable, but simultaneous loss of both genes is lethal to the cell. The discovery of SL relationships in gene pairs can reveal cancer-mechanism-related targets for the development of therapeutic drugs. A classic example in translational cancer research is the SL relationship between *BRCA* and *PARP*, which control two parallel paths in the DNA damage pathway. The discovery of this relationship led to the development of the first *PARP* inhibitor and first SL-based Food and Drug Administration (FDA)-approved therapy for use in the clinic, niraparib [1], which is used to treat patients with breast and ovarian cancers with *BRCA* somatic mutations. Mechanistically, in tumor cells with *BRCA* mutations, only *PARP* is active in the DNA damage pathway, and niraparib can completely shut down the pathway and eliminate tumor cells. On the other hand, in normal cells, both *BRCA* and *PARP* are active in the DNA pathway, so blocking *PARP* alone has little effect on cell viability.

High-throughput gene-combination CRISPR double-knockout (CDKO) technologies, including combinatorial RNA interference (RNAi) screening [2] and the combinatorial CRISPR-Cas9 technique [3–7], have been developed to identify SL gene pairs. However, unlike single-gene-based genome-wide screening technologies [8], CDKO technologies can only screen several hundred genes and their combinations. Therefore, computational models are needed to predict and select SL gene pairs for CDKO experimentation. The conduct of a CDKO experiment in an individual cancer cell requires SL prediction to be for individual cancer cell as well.

## Related works

Computational models developed to predict SL can be broadly categorized as rule-based statistical inference methods, network-based methods, and machine learning methods [9]. Rule-based statistical inference methods, such as DAISY [10], ISLE [11], ASTER [12], and MiSL [13], rely on predefined synthetic lethality concepts and conduct statistical tests using pan-cancer multi-omics data from both clinical tumor samples and cancer cell lines to identify SL gene pairs. Network-based approaches, such as VIPER [14], OptiCon [15], and Double-target selection guided by CRISPR screening and network (DSCN) [16] rank gene combinations based on the proportion of the gene regulatory network they control. Similar to rule-based statistical inference, network approaches use pan-cancer multi-omics data. Neither rule-based statistical inference nor network approaches use the SL experiment data in model development. In contrast, machine learning methods rely on SL experiment data in model development and SL prediction. Early machine learning methods include random forest (RF) [17–19], support vector machine (SVM) [20], ensemble-based models [21–23], and matrix factorization methods [24–26]. In recent years, deep learning methods, especially graph neural network (GNN) methods, were utilized for SL prediction. These methods integrate graph topology developed from biological pathways, gene networks, and gene associations with diseases and drugs. Deep learning methods have shown much better SL prediction than machine learning approaches; few examples include dual-dropout graph convolutional network (DDGCN) [27], graph contextualized attention network for synthetic lethality (GCATSL) [28], knowledge graph for synthetic lethality (KG4SL) [29], pair-wise interaction learning-based graph network for synthetic lethality (PiSL) [30], and multi-view graph convolution network for individual sample based synthetic lethality prediction (MVGCN-iSL) [31].

However, the capacities of all these models to predict SL are limited by their approaches to SL experiment design and SL discovery that ignore cell context, thereby preventing generalization of their findings. All these approaches (except MVGCN-iSL) were proposed to predict SL at the population level. Both rule-based statistical inference and network approaches predict pan-cancer SL gene pairs and so can most machine learning methods by gathering data from the SynLethDB 2.0 database without explicitly considering cancer cell–specific biology contexts [32]. However, experimental evidence suggests that SL in cancer is more heterogeneous than homogeneous [33]. One CDKO study in three cancer cells, A549, HeLa, and 293T cell lines [34], revealed no overlapping SL gene pairs among 2628 gene pairs, and another study on two cancer cells, K562 and Jurkat, showed overlap of only 0.074% (82 SL) gene pairs among 111 156 possible gene pairs. Ideally, SL prediction models need to be trained using cell-specific SL data.

DDGCN, PiLSL, GCATSL, KG4SL, and MVGCN-iSL represent five high-performing GNNs in SL prediction. DDGCN utilized graph convolution, modeling a gene’s neighboring genes and their SL relationship to predict new SL relationships. PiLSL developed one step further than DDGCN, characterizing the neighboring SL genes between two genes to predict their SL relationship. GCATSL employs attention mechanisms to aggregate local and global node representations of networks, KG4SL uses multiple entity and relationship types in SynLethDB’s knowledge graph, and MVGCN-iSL utilizes multiple gene networks in obtaining gene representations across multiple molecular signaling pathway datasets. GCATSL, KG4SL, and MVGCN-iSL methods rely on biological networks, such as BioGRID [35], and predict SL from the topological patterns of biological networks, which demonstrates a tendency toward overtraining or potential bias toward training samples [36].

In developing SL prediction models, training and test sets of gene pairs are prepared in two scenarios. (i) Overlap (O): gene pairs are randomly selected into training and test sets, and two gene sets have overlapping genes. (ii) Nonoverlap (NO): training and test sets have no overlapping genes. It is known that almost all methods performed much worse in the NO test set than O test set. Using the area under the receiver operating characteristic curve (AUC-ROC) as the criteria of SL prediction performance, DDGCN has 0.54 [NO] versus 0.86 [O], PiLSL has 0.67 [NO] versus 0.95 [O], GCATSL has 0.56 [O] versus 0.91 [NO], and KG4SL has 0.50 [O] versus 0.94 [NO] [30]. These literature review suggests that current SL prediction models cannot predict well on SL for new genes. In other words, the SL among genes in the train set couldn’t be generalized to SL among new genes in the test set.

**Figure 1:**
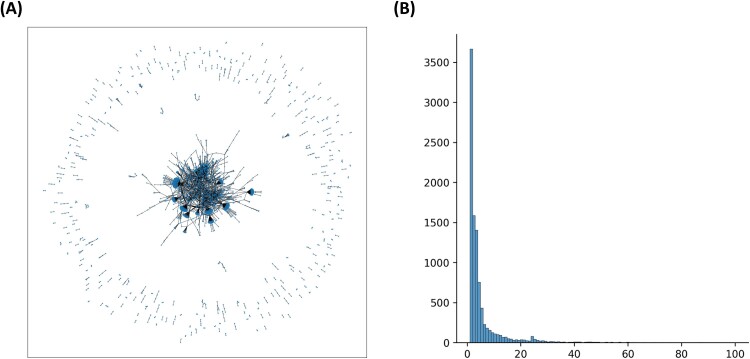
SL connectivity pattern in the SynLethDB. (A) SL graph in which each node is one gene, and each edge represents SL relationship. We randomly sampled 3000 edges to clarify visualization. (B) Histogram of node degree. Here, the node degree represents the number of SL partners of each gene.

To seek for scalable properties of SL gene pairs, we investigated SL patterns utilizing SynLethDB 2.0, a comprehensive database comprising information for 36 402 human SL pairs [32]. We observed the greater likelihood of genes occurring in SL pairs than those in non-SL pairs (Fig. 1). We found that the SL network has a scale-free [37] distribution, which reflects some gene nodes having more connections than other nodes. Considering the possibility of selection bias in genes included in the SynLethDB database [38], we investigated two unbiased cancer cell–specific SL datasets from a gene combination double-knockout (CDKO) experiment encompassing the pairwise interactions of 472 genes (Methods) [39]. Both exhibited similar scale-free patterns to those noted in the SynLethDB database (Supplementary Fig. 1). This SL data exploration motivated us that SL connectivity, i.e. the number of SL partners, can be a scalable property shared among SL gene pairs, extending to out-of-sample genes. If there is a good SL connectivity prediction model developed in train set, it can be generalizable to new genes in test set.

Therefore, in this paper, we propose a two-step multilayer encoder SL prediction model for individual cancer cell (MLEC-iSL). In the first step, a deep learning model is developed to predict a gene’s SL connectivity, which comprises gene, graph, and transformer encoders. Then, in the second step, the SL between two genes is predicted by their predicted SL connectivities. Because SL connectivity is scalable, we hypothesize that this two-step method leads to a better prediction performance than the other existing SL prediction models. Furthermore, in addition to published CDKO experiment data, we generate two rounds of CDKO experiments in the 22Rv1 cell line to first train and then evaluate the model. For the first time, CDKO experiments were purposely designed and carried out for validating SL prediction model performance.

## Materials and methods

### Data collection for MLEC-iSL model development and prediction evaluation

#### Synthetic lethality data in K562 and Jurkat cells

We obtained cancer cell–specific CDKO data using the mapping system reported by Horlbeck *et al*., who utilized a combinatorial CRISPR technique to screen pairwise genetic interactions of 472 genes in two leukemia cell lines (K562 and Jurkat) [39]. Based on Horlbeck’s SL score, we labeled gene pairs with genetic interaction scores below −3 as positive SL samples. In the K562 cell line, 1523 of 100 128 gene pairs (1.5%) were SL, whereas in the Jurkat cell line, only 373 of 74 691 gene pairs (0.5%) were SL. We calculated the SL connectivity of each gene as the number of positive SL partners and utilized this number as the intermediate objective for regression task.

#### Multi-omics data

We downloaded gene expression, copy number, and mutation profiles from the Cancer Cell Line Encyclopedia (CCLE) database [40] and obtained CRISPR essentiality data from the Cancer Dependency Map portal (DepMap) [41]. Our work considered both population-based multi-omics, consisting of omics profiles across all cell lines, and cell-specific multi-omics, which correspond to omics profiles of K562 and Jurkat. For mutation data, we counted the number of mutations in each gene specific to each cell line as the input.

#### Biological networks data

We collected both physical protein–protein interactions and genetic interactions from BioGRID database [35] and an integrated pathway network built by OptiCon [15], which includes three high-quality pathway databases—Reactome [42], Kyoto Encyclopedia of Genes and Genome (KEGG) [43], and the National Cancer Institute’s Nature Pathway Interaction Database [44]. For genetic interactions from BioGRID, we removed gene pairs present in K562 and Jurkat data from Horlbeck *et al*. [39] to avoid data leakage. To make these networks specific to the cell line of interest, we removed gene nodes not expressed in the specific cell line.

### Setup of computational experiments

Comparison of multiple machine learning methods involved individual computational experiments for Jurkat and K562 cancer cells. In each experiment, we randomly split the dataset into an 80% training set and a 20% held-out external test set, and we selected hyper-parameters based on a 20% internal validation set from the training set. In particular, training and test data split was performed in a nonoverlap setting, i.e. no overlap genes between test set and training set. The test set is also called hold-out test set in the text. Default hyper-parameters were used for training and evaluating the comparison methods. In order to reduce the potential bias in each individual test/training set split, model performance is evaluated on 10 repeated held-out test sets (Supplementary Methods).

#### Evaluation metrics

We consider three evaluation metrics to compare SL prediction performance. The first two metrics, AUC-ROC and area under the precision–recall curve (AUPR), are threshold-free. The third metric, Precision@k, measures the proportion of true-positive samples in the top k% of predictions by the model to demonstrate the model’s ability to prioritize the top SL pairs. We report measurements averaged among 10 repeated runs.

### Architecture of the MLEC-iSL model

**Figure 2:**
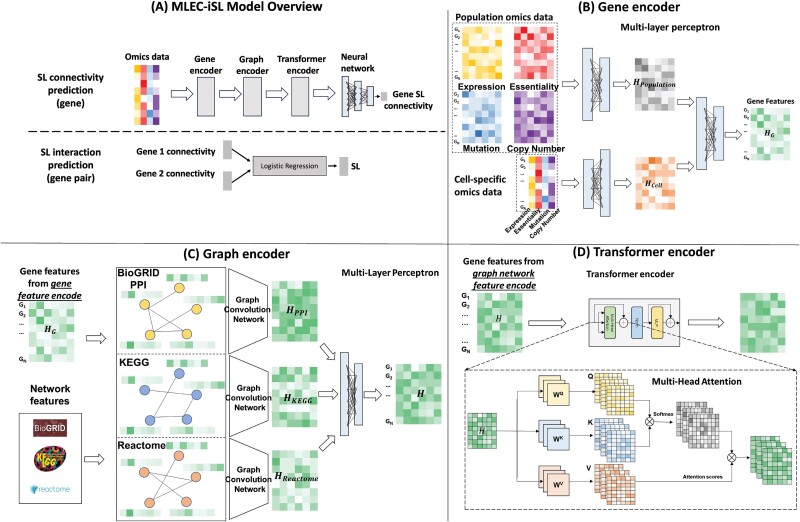
MLEC-iSL model architecture. (A) Overview of the model. (B) Gene encoder architecture, where population and cell-specific omics are encoded. (C) Graph encoder architecture, where network features and output of (B) are encoded. (D) Transformer encoder, where output of (C) is analyzed through a global topology lens.

Our proposed MLEC-iSL model is a two-step model, built in Pytorch [45] and PyG [46]. It is designed to predict SL connectivity first. Then, SL between two genes is predicted by their predicted SL from the first step. The model comprises four components—a gene feature encoder, graph encoder, transformer encoder, and predictor from SL connectivity to SL (Fig. 2, Supplementary Methods).

In the first step, the model is trained for SL connectivity task using gene pairs’ actual SL connectivities, i.e. the number of their SL partners, in a regression task. First, the gene encoder module takes in gene omics features, both population-based and cell-specific, encoding them separately and integrating them to a final gene representation matrix. Second, a multiview graph encoder integrates gene encoder’s generated features with network features, leveraging local topology. Third, transformer encoder extends model training from local topology to global topology through the use of multihead attention, considering all possible pairwise associations between the nodes. Finally, a multilayer perceptron (MLP) is used to predict SL connectivity from transformer encoder–generated features.

In the second step, a logistic regression model is trained to predict for SL interactions using SL connectivities of gene pairs, in a classification task. During training, the regression model uses the predicted SL connectivities from the first part of MLEC-iSL to predict for SL.

We implemented MLEC-iSL model using Pytorch [45] and PyG packages in Python [46]. Adam optimizer is used in training, with a low learning rate of 5e-5 to prevent overfitting [47], and updating parameters via gradient descent [48]. Dropouts between encoder and predictor layers are set to 50% and 70%, respectively. To ensure model convergence, a high number of training epochs (default: 1000) and early stopping epochs (default: 200) are chosen. We conduct all experiments in Ohio Supercomputer Center (OSC) using two NVIDIA® Tesla V100 GPUs with 32GB RAM.

### CRISPR double-knockout experimental validation of MLEC-iSL prediction and synthetic lethality selection

To demonstrate the performance of MLEC-iSL prediction and the selection of SL gene pairs, MLEC-iSL was initially trained in the data from the first CDKO experiment in the prostate cancer cell line, 22Rv1. SL and non-SL gene pairs were then predicted and selected for the second CDKO experiment in the same 22Rv1 cell. The second CDKO experiment differs from the first experiment in two ways: gene pair targets do not cover all pairwise combinations, and gene targets are not included in the first experiment (i.e. out-of-sample). While the first experiment targeted 50 genes and their all possible 1225 gene pairs, the second experiment targeted 151 genes and their 987 specific gene pairs. The process of MLEC-iSL model development and experiment validation is outlined in Supplementary Methods.

## Results

### Multi-omics data and biological networks contribute to synthetic lethality prediction

Our model incorporated three types of input feature−population-based multi-omics, cell-specific multi-omics, and multiple biological networks. We examined the contribution of each feature type to our model’s performance (Table 1A) and then investigated the detailed importance of each biological network and single population–based omics data (Table 1B). All experiments were performed on the K562 cell line under nonoverlap setting (Materials and Methods).

**Table 1:** Ablation analysis of features and encoder modules.

(A) Ablation of input feature types					
Network features	Population-based omics	Cell-specific omics	AUPR	AUC-ROC	Precision@10
−	−	−	0.505 $\pm$ 0.041	0.503 ± 0.042	0.446 ± 0.167
+	−	−	0.565 ± 0.062	0.557 ± 0.052	0.623 ± 0.141
−	+	−	0.598 ± 0.043	0.597 ± 0.043	0.654 ± 0.123
+	+	−	0.707 ± 0.031	0.706 ± 0.024	0.807 ± 0.091
+	+	+	0.719 ± 0.021	0.727 ± 0.025	0.819 ± 0.081
**(B) Ablation of individual features**					
**Feature type**			**AUPR**	**AUC-ROC**	**Precision@10**
PPI-physical			0.603 ± 0.038	0.601 ± 0.032	0.662 ± 0.116
PPI-genetic			0.670 ± 0.039	0.693 ± 0.040	0.723 ± 0.083
Pathway			0.685 ± 0.036	0.679 ± 0.032	0.808 ± 0.075
Expression			0.693 ± 0.039	0.699 ± 0.023	0.801 ± 0.110
Essentiality			0.708 ± 0.037	0.703 ± 0.028	0.816 ± 0.141
CNV			0.586 ± 0.029	0.575 ± 0.025	0.697 ± 0.092
Mutation			0.513 ± 0.056	0.508 ± 0.069	0.515 ± 0.150
**(C) Ablation of encoder modules**					
**Encoder module type**			**AUPR**	**AUC-ROC**	**Precision@10**
Graph			0.622 ± 0.031	0.612 ± 0.029	0.731 ± 0.112
Graph + Transformer			0.719 ± 0.021	0;.727 ± 0.025	0.819 ± 0.081

Notes that “-“ indicates not used, and “+” represents used. The numbers in each cell are the average performance across 10 repeated runs.

As Table 1A shows, compared to random features, the use of biological networks and population-based omics features contributed to the prediction of SL, with AUPR increasing from 0.505 (random features) to 0.565 (biological networks) and 0.598 (population-based omics features). AUC-ROC and Precision@10 followed similar patterns. Surprisingly, the combined use of biological networks and population-based omics features improved AUPR from 0.505 to 0.707, indicating a synergistic effect of their combined use. The inclusion of the cell-specific multi-omics features, in addition to population-based omics and network features, also contributed slightly to the model’s performance.

Investigating the contributions of different types of biological networks to SL prediction, we found that the physical protein-protein interaction (PPI) network provided the least gain over a random network, whereas genetic interactions contributed greatly to prediction. Importantly, the pathway network showed substantial importance in predicting SL, with AUPR of 0.685 and Precision@10 of 0.808, demonstrating the essential role of systems biology in the study of SL. Comparison of each type of population-based omics feature with random feature input demonstrated the poorest predictive performance of the mutation feature, which may be attributable to its sparse representations, and copy number variation did not perform much better. Notably, expression and essentiality contributed significantly to SL prediction, with AUPR increasing from 0.565 (random) to 0.693 (expression) and 0.708 (essentiality). For simplicity, we therefore chose to consider only population-based expression and essentiality in our final model. Overall, each feature contributed uniquely to the prediction, and the integration of features performed best, justifying the importance of including multiple sources of features.

### Incorporation of a transformer encoder greatly improves synthetic lethality prediction

A major innovation in our proposed model is the incorporation of a transformer encoder to learn long-range dependencies. Here, we investigated whether its inclusion could improve predictive performance. We compared our graph transformer module (graph encoder + transformer encoder) with a graph-encoder-only module, with input features and other components being fixed (Table 1C). All experiments were performed on the K562 cell line under nonoverlap setting.

The use of a transformer encoder stacked on top of a local topology–aware graph encoder greatly outperformed the model with only a graph encoder, with increases in AUPR from 0.622 to 0.719, AUC-ROC, from 0.612 to 0.727, and Precision@10, from 0.731 to 0.819. This result revealed that the global topology of biological networks is predictive of SL patterns and that the transformer encoder succeeded in learning long-range dependencies in the network.

### MLEC-iSL achieves state-of-the-art synthetic lethality prediction

**Figure 3:**
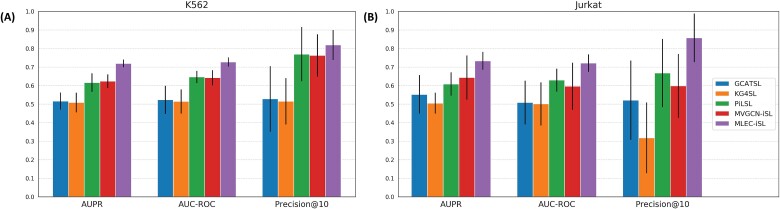
Model comparisons for nonoverlap evaluation. (A) Comparison results for the K562 cell line. (B) For peer review, comparison results for the Jurkat cell line.

We compared prediction performance among our proposed MLEC-iSL and four existing GNN-based methods with demonstrated state-of-the-art performance—GCATSL, KG4SL, PiLSL, and MVGCN-iSL. We used two cell-specific datasets (K562 and Jurkat) to compare AUPR, AUC-ROC, and Precision@10 metrics under nonoverlap evaluation setting (Fig. 3), which measures the ability of a model to generalize to novel genes.

The poor performance of GCATSL and KG4SL in both datasets across all metrics indicated their inability to achieve inductive learning for genes not included in the training set. Using omics features and a special model design, PiLSL achieved acceptable performance in the two datasets, with as high as 0.692 in the Precision@10 metric when evaluated using the K562 cell line. PiLSL’s performance dropped slightly when evaluated on the Jurkat cell line, and MVGCN-iSL performed similarly to PiLSL. When evaluated on the K562 cell line, our model outperformed all four methods, with AUPR of 0.719, AUC-ROC of 0.727, and Precision@10 of 0.819, and better results on the Jurkat dataset demonstrated the model’s highly robust performance. Similar results were observed on the precision–recall curves (Supplementary Fig. 2).

### MLEC-iSL prediction and synthetic lethality selection are validated in two rounds of gene-combination double-knockout experiments

To evaluate the performance of MLEC-iSL in a different cancer cell, we conducted two CDKO experiments in 22Rv1, a prostate cancer cell. In the first 22Rv1 CDKO experiment [49], we targeted 50 genes and their 1225 gene pairs. Using 10 different SL scores presented in the synthetic lethality knowledge base (SLKB) [50], we determined 88 pairs to be SL and 1137 to be non-SL (Supplementary Methods). The MLEC-iSL model was trained using this data set and achieved cross-validation performances of 0.67 AUC, 0.71 AUPR, and 0.83 for Precision@10.

We then used MLEC-iSL to make SL predictions for gene pairs in apoptosis, mitotic cell death, and autosis pathways from KEGG and XDeathDB databases [43, 51]. The MLEC-iSL model predicted SL probabilities among 151 unique genes of interest in three pathways and their 11 345 unique combinations. One hundred and forty-two out of 151 genes were not present in the first CDKO experiment. Afterward, we selected 987 of these 11 345 gene pairs to validate the model’s predictions by a second CDKO experiment (Materials and Methods, Supplementary Tables 1 and 2). These selected gene pairs were further proportionally distributed among six combinations of three cell death pathways, based on the size of pathways (Supplementary Table 3). Our MLEC-iSL predicted 517 among 987 gene pairs to be SL and 470 to be non-SL (Materials and Methods, Table 2).

**Table 2:** Gene pair selection, SL validation, and prediction evaluation.

Pathways between a gene pair	Predicted SL	Predicted non-SL	Validated SL	Validated non-SL	Precision	Recall	AUC-ROC	AUPR	Precision@10	SL Ratio
Apoptosis/Apoptosis^a^	350	300	295	355	0.41	0.49	0.451	0.425	0.446	0.454
Apoptosis/Autosis^b^	20	20	7	33	0.25	0.71	0.630	0.352	0.500	0.175
Apoptosis/Mitotic^c^	80	80	84	76	0.48	0.45	0.450	0.522	0.563	0.525
Mitotic/Mitotic^d^	50	50	67	33	0.64	0.48	0.466	0.711	0.700	0.670
Autosis/Mitotic^e^	16	20	9	27	0.25	0.44	0.500	0.279	0	0.250
Autosis/Autosis^f^	1	0	0	1	0	–	N/A	0	0	0
Total	517	470	462	525	0.43	0.48	0.415	0.424	0.418	0.468

Note: The threshold of SL/non-SL prediction probability among gene pair cell death pathways are: ^a^0.78 in apoptosis/apoptosis, ^b^0.80 in apoptosis/autosis, ^c^0.44 in apoptosis/mitotic, ^d^0.08 in mitotic/mitotic, ^e^0.50 in autosis/mitotic, and ^f^0.70 in autosis/autosis. The gene pairs that scored higher than the threshold were predicted as SL and non-SL otherwise.

After the experiment, 10 SL scores were calculated for all 987 gene pairs (Supplementary Table 4). To remain consistent in calling SL gene pairs in both first and second CDKO experiments, we selected the same 3% threshold scores in first experiment and applied it to the second experiment (Supplementary Table 5). As a result, each scoring algorithm predicted the top number of SL gene pairs based on the thresholds they had. We thus validated 462 among these 987 selected gene pairs as SL and determined 525 to be non-SL, a 46.8% SL ratio (Table 2). The overall SL prediction performance in this 22Rv1 experiment was 0.415 AUC, 0.424 AUPR, and 0.418 Precision@10 (Table 2). Gene pairs among the mitotic cell death/mitotic cell death pathways achieved the highest prediction with 0.466 AUC-ROC, 0.711 AUPR, and 0.700 Precision@10. Comparing two CDKO experiments in 22Rv1, the fraction of SL gene pairs goes from 88/1225 = 7.2% in the first experiment, to 462/987 = 46.8% in the second experiment, *P* value <.0001 in chi-square test. This significant increase in detecting SL gene pairs is primarily attributed to MLEC-iSL’s gene pair selection in the second CDKO experiment, while genes are randomly paired in the first CDKO experiment.

### Synthetic lethality gene pairs and dependency between apoptosis and mitosis pathways in the 22Rv1 cell line

**Figure 4:**
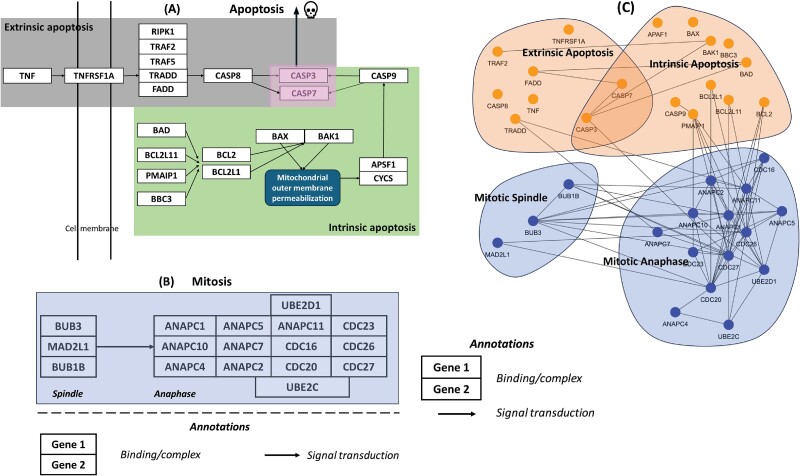
Apoptosis and mitotic cell death pathways in 22Rv1 SL CDKO experiment. (A) Targeted extrinsic and intrinsic apoptosis genes in CDKO. (B) Targeted anaphase and spindle mitotic cell death genes in CDKO. (C) Validated SL gene pairs across apoptosis and mitotic cell death pathways.

Apoptosis has two well-established pathways—extrinsic and intrinsic (Fig. 4A). Extrinsic apoptosis is initiated when the tumor necrosis factor (TNF) binds to TNF receptor superfamily member 1A (*TNFRSF1A*) [52], leading to the assembly of a signaling complex that includes *TRADD*, *FADD*, *TRAF2*, *TRAF5*, and *RIPK1* [53, 54]. The central process of intrinsic apoptosis is mitochondrial outer membrane permeabilization (MOMP), which is controlled by the apoptosis regulators *BAX* and/or *BAK1*.

The mitosis pathway (Fig. 4B) includes a spindle assembly checkpoint and anaphase-promoting complex. The spindle comprises *BUB1B*, *BUB3*, and *MAD2L1*. In the subsequent anaphase, this protein complex interacts with the other protein complex, which includes seven ANAPC proteins and five CDC proteins, triggering cell death [55–57].

In our second-round CDKO experiment in 22Rv1 cells, we observed six of seven SL gene pairs between the extrinsic and intrinsic apoptosis pathways*.* This is the first major discovery deserving more studies in other cells. The second discovery is a strong SL connectivity between mitosis anaphase and intrinsic apoptosis. Among 14 SL gene pairs between the apoptosis and mitosis pathways, 12 SL gene pairs were between intrinsic apoptosis and mitosis anaphase (Fig. 4C).

### The performance of synthetic lethality hub genes in the synthetic lethality network

**Figure 5:**
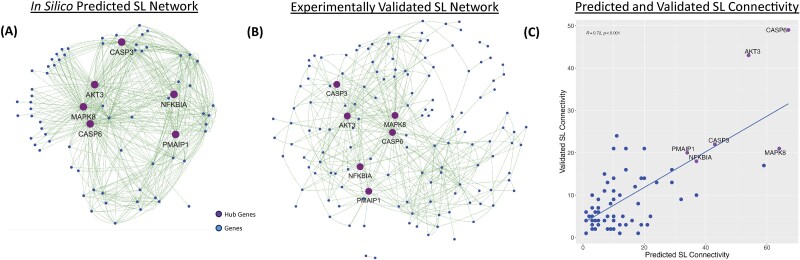
MLEC-iSL’s synthetic lethality prediction and CDKO synthetic lethality evaluation networks. (A) for peer review and hub genes for 22Rv1 cell line. (C) SL connectivity of overlapping genes between predicted and validated SL networks (Pearson coef. 0.72, *P*-value <.01). In (A–C), purple genes denote the 6 overlapping hub genes among top 10 genes of prediction and evaluation groups.

To observe SL connectivity’s scalable property, we also closely examined the highly connected genes in the SL network, i.e. hub genes, among both predicted SL gene pairs by MLEC-iSL and validated SL gene pairs in the CDKO experiment. Among the top 10 hub genes in both predicted and validated SL pairs, we observed six of the hub genes to be overlapping—*AKT3*, *CASP3*, *CASP6*, *MAPK8*, *NFKBIA*, and *PMAIP1*. Figure 5A presents the SL network predicted by MLEC-iSL and Fig. 5B, which was validated in the CDKO experiment. Their SL hubs appear very similarly consistent as well (Fig. 5C).

## Discussion

In this paper, we proposed MLEC-iSL, a novel two-step multilayer encoder model for individual sample-specific SL prediction, which incorporates multiple biological networks and multi-omics data for SL connectivity and interaction prediction. The primary contributions of our work are the introduction of the concept of SL connectivity as an intermediate learning objective to improve the generalization and interpretability of SL findings and the incorporation of transformer encoder to enable the learning of long-range dependencies in the networks. Our method outperformed existing state-of-the-art deep learning methods by a large margin and showed outstanding generalization ability to predict for out-of-sample genes.

This paper is the first work that CDKO validation is purposed designed and carried out for a SL prediction model. MLEC-iSL guided CDKO experiment also validated 6 of the 10 predicted SL gene hubs and yielded a high correlation (Pearson coef. = 0.72, *P*-value <.01). The experiment further revealed more interesting SL gene pairs and dependency between apoptosis and mitotic cell death pathways. These data strongly support our hypothesis that SL connectivity, the underlying assumption of MLEC-iSL, leads to better SL prediction performance.

Several challenges remain. First, the deficiency of individual sample–specific features prevents the model from learning more sample-specific SL patterns. In the future, more sample-specific data can be collected from single-cell omics data as well as perturbation data that might reflect more detailed sample-specific characteristics. Second, our proposed graph transformer architecture employs a simple method to integrate the GNN and transformer, and the design of more complicated interactions between them can improve performance [58]. Third, the availability of sample-specific data limits the from-scratch development of specific machine learning models. Investigating transfer learning to borrow information from data-rich samples to train the model for data-poor samples is a promising future direction.

**Key Points**

- We recognize and utilize synthetic lethality (SL) connectivity, i.e. the number of SL partners of a gene, as a scalable SL property in SL prediction.
- Our proposed multilayer encoder for individual sample-specific SL prediction model (MLEC-iSL) achieves state-of-the-art performance in out-of-sample gene pairs.
- MLEC-iSL guided CRISPR-Cas9 double knockout (CDKO) experiment achieves a 46.8% SL rate (462/987 gene pairs), compared to a 7.2% SL rate CDKO experiment (88/1225 gene pairs) without guidance from MLEC-iSL.
- This paper is the first work that *in vitro* validation is purposely designed and carried out for an *in silico* SL prediction model.

## Supplementary Material

Supplemental_Data_bbae425

## Data Availability

The source code and processed data used for model evaluations for our MLEC-iSL pipeline are freely available on GitHub (https://github.com/kunjiefan/MLEC-iSL). Raw reads and aligned counts can be accessed under project GSE262953 at Gene Expression Omnibus.
