# Supplementary material for: Synthetic lethal connectivity and graph transformer improve synthetic lethality prediction
Source: Brief Bioinform. 2024 Aug 30;25(5):bbae425. doi: 10.1093/bib/bbae425 (PMC11361842; doi:10.1093/bib/bbae425)
Supplement: Supplemental_Data_bbae425 [file supplemental_data_bbae425.zip › Supplementary Methods - Revised.docx]

**SUPPLEMENTARY METHODS**

## **Setup of computational experiments to evaluate model performance**

*Selection of training and test sets*. Data of SL pairs can be split into training and test sets by leaving out either a gene combination or all pairs of a gene, leading to overlap and non-overlap data splits. Under overlap setting, the sharing of genes by gene pairs in the training and test sets can lead to the model’s easily overfitting to genes with many SL partners, yielding superficially high-performance results. In non-overlap setting, the absence of genes in the test set from those in the training set allows measurement of the model’s ability to generalize to the SL of novel genes. In this work, we focus on non-overlap evaluation setting when we perform ablation analysis and compare our model with other methods.

*Comparison of methods*. We compared our model with four state-of-the-art GNN-based methods−GCATSL [1], KG4SL [2], PiLSL [3], and MVGCN-iSL [4]. We used the default hyper-parameters specified by those authors and re-trained their models using the same data splits we used in our model.

**Architecture of the MLEC-iSL model**

## *Gene encoder.* Input gene omics features are either population-based, including gene expression and essentiality profiles across multiple cell lines that might help reflect common synthetic lethality mechanisms across cancer types, or cell-specific features that contextualize a specific cancer cell; including gene expression, essentiality, copy number variation, and mutation.

These two types of features are first encoded separately and then integrated into a final gene representation matrix by three multi-layer perceptron (MLP) models as:

|  | $\boldsymbol{H}_{p}=MLP ([\boldsymbol{X}_{\text{exp}}\oplus\boldsymbol{X}_{\text{ess}}\oplus\boldsymbol{X}_{\text{mut}}\oplus\boldsymbol{X}_{\text{cnv}}]),$ | *(1)* |
| --- | --- | --- |
|  | $\boldsymbol{H}_{c}=MLP ([\boldsymbol{v}_{\text{exp}}\oplus\boldsymbol{v}_{\text{ess}}\oplus\boldsymbol{v}_{\text{mut}}\oplus\boldsymbol{v}_{\text{cnv}}]),$ | *(2)* |
|  | $\boldsymbol{H}_{G}=MLP ([\boldsymbol{H}_{p}\oplus\boldsymbol{H}_{c}]),$ | *(3)* |

in which each MLP is a fully connected feedforward neural network composed of multiple perceptron layers. Each layer is defined as $\sigma(\boldsymbol{WX}+\boldsymbol{b})$, in which $\boldsymbol{X}$ is the input, $\boldsymbol{W}and\boldsymbol{b}$ are the learnable weights, and $\sigma$ is an activation function, such as $ReLU (\cdot)=max (\cdot, 0)$. $\boldsymbol{X}_{\text{exp}}$, $\boldsymbol{X}_{\text{ess}}$, $\boldsymbol{X}_{mut}$ and $\boldsymbol{X}_{cnv}$ are four CCLE profile matrices (expression, essentiality, mutation, and copy number variation); $\boldsymbol{v}_{\text{exp}}$,$\boldsymbol{v}_{\text{ess}}$, $\boldsymbol{v}_{mut}$ and $\boldsymbol{v}_{cnv}$ are four cell-specific omics feature vectors for an individual cancer cell; and $\oplus$ is the concatenation operation.

*Graph encoder.* We consider three biological networks for the prediction of SL connectivity−the physical protein-protein interaction and genetic interaction networks and an integrated signaling pathway network. To model multiple biological networks along with gene features, we propose a multi-view graph encoder based on graph convolution operation [5] that basically computes a node’s new features as the weighted average of the node’s own features and those of its local neighbors. This neighborhood-local aggregation utilizes relational inductive bias encoded by the graph’s topology. In each input network (i.e., view), we adopt a two-layer graph convolutional network (GCN) to model the relations among genes defined by the local topology to generate network-specific representation. An MLP is then used to integrate these network-specific representations into one unified representation matrix:

|  | $\boldsymbol{H}_{i}=f (\boldsymbol{A}_{i}f (\boldsymbol{A}_{i}\boldsymbol{H}_{G}\boldsymbol{W}_{i}^{0})\boldsymbol{W}_{i}^{1})\text{,}\text{ }\text{i}\text{=}\left\{ \text{phy}\text{, gen, path} \right\},$ | *(4)* |
| --- | --- | --- |
|  | $\boldsymbol{H}=MLP ([\boldsymbol{H}_{phy},\boldsymbol{H}_{gen},\boldsymbol{H}_{path}]),$ | *(5)* |

where $\boldsymbol{A}_{phy}$, $\boldsymbol{A}_{gen}$, $\boldsymbol{A}_{path}$ are symmetrically normalized adjacency matrices of the physical protein-protein interaction (*phy*), genetic interaction (*gen*), and pathway (*path*) networks, and f is an activation function, e.g., $ReLU (\cdot)=max (\cdot, 0)$.

## *Transformer encoder.* The inability of graph convolution to model the association between a gene node and its distal gene nodes causes graph convolution of a gene node to be restricted to its local neighborhoods. We therefore propose the use of a transformer encoder to model all pairwise associations among all gene features to elucidate relationships between nodes that are far apart in the network [6]. Application of the transformer encoder to gene features generated from the multi-view graph encoder that already takes local topology into account, thereby extending model learning from the local topology to global topology. This process enhances the model’s systems biology understanding of SL and SL connectivity. The combined graph and transformer encoder is a graph transformer module.

The core part of a transformer encoder is a self-attention module that calculates a weighted average of feature representations with the weight proportional to the similarity between pairs of representations. Basically, each feature vector is transformed into a set of three vectors−query, key, and value vectors−and a dot product is used to calculate the associations (attention scores) among feature elements in the transformed space based on the query and key vectors. To measure similarity among multiple representation subspaces, each feature vector is transformed into multiple sets of query, key, and value vectors to calculate multiple attention scores. This is termed multi-head attention. The complete transformer encoder is defined as:

|  | $Attention\left( Q,K,V \right)=softmax\left( \frac{QK^{T}}{\sqrt{d}} \right)V,$ | *(6)* |
| --- | --- | --- |
|  | $\text{hea}\text{d}_{i}=Attention\left( QW_{i}^{Q},KW_{i}^{K},VW_{i}^{V} \right),$ | *(7)* |
|  | $\mathbf{H}_{T}\boldsymbol{=}MLP (\left\{ {}_{i=1}^{K}\left\Vert{head}_{i} \right. \right\}W^{O})$ | *(8)* |

where $Q=K=V=\mathbf{H}$, and *d* is the feature input dimension. Softmax denotes a row-wise softmax normalization function in which each row sums to one; $W_{i}^{Q}$, $W_{i}^{K}$, and $W_{i}^{V}$ are parameter matrices that transform the input into $i^{th}$ subspaces; $W^{O}$ is a parameter matrix that integrates representations from multiple heads; and $\parallel$ is the concatenation operator.

## *Predictor and optimization.* With gene representation generated by the transformer encoder, we use a three-layer perceptron model to predict the SL connectivity for each gene. Considering the prediction of SL connectivity as a regression task, we use the mean squared error (MSE) as the loss function for optimization:

|  | $\boldsymbol{z}=MLP (\boldsymbol{H}_{T})$ | *(9)* |
| --- | --- | --- |
|  | $\mathcal{L=}\Phi(\boldsymbol{z},\boldsymbol{y})$ | *(10)* |

where $\Phi$ is the MSE function, and $\boldsymbol{y}$ denotes the ground truth SL connectivity vector. To further predict SL between gene pairs, a logistic regression model is used with the predicted SL connectivity of each gene in the pair as input features.

**CDKO experimental validation of MLEC-iSL prediction and synthetic lethality selection**

*The first CDKO experiment in 22Rv1*. The first experiment [7] targeted 50 genes and their 1,225 gene combinations. Given the diversity among SL calculation algorithms, we chose all scoring algorithms implemented in the synthetic lethality knowledge base (SLKB), a recently developed database for SL data from CDKO experiments [8]. SLKB implements five unique scoring algorithms, namely Median-B/NB, sgRNA-B/NB, Horlbeck, GEMINI, and MAGeCK scores. Based on these five methods, we have employed SLKB’s ten different scores: Median-B/NB with and without standard error standardization, sgRNA-B/NB, Horlbeck, GEMINI SL strong, and MAGeCK with and without standard error standardization. Gene pairs were ranked based on their scores generated using each algorithm. A low score indicates strong SL in every method except the GEMINI score, where a high score denotes strong interaction. Using the first CDKO experiment data in 22Rv1, we obtained the top 3% of the gene pairs from each score and labeled the gene pairs scored by at least two methods as SL. As a result, 88 gene pairs were identified as SL and remaining 1,137 pairs were identified as non-SL.

*The MLEC-iSL model was trained in this first CDKO experiment dataset.* Similar to the training of MLEC-iSL on K562 and Jurkat cell lines, we applied a non-overlap split to this dataset, in which all genes were split into three gene sets with a 70% train/10% validation/20% test split and obtained the gene pairs within each split. From the same multi-omics sources, 22Rv1’s omics were used in the model’s training.

*The selection of synthetic lethal and non-synthetic lethal gene pairs based on the MLEC-iSL model, cell death pathways, and hub genes.* The trained MLEC-iSL model provided prediction probabilities for genome-wide SL gene pairs. To discover more interpretable SL gene pairs and validate the model’s prediction performance, we utilized two strategies in selecting genes and gene pairs. First, we wanted to understand SL connections in three cell death pathways−autosis, apoptosis, and mitotic cell death. Genes in the apoptosis pathway were curated from KEGG [9], and those in the apoptosis and mitotic cell death pathways were curated from XDeathDB [10], yielding a total of 151 genes, 131 in the apoptosis pathway, 18 in the mitotic cell death pathway, and two in the autosis pathway. For the 151 genes, MLEC-iSL predicted a total of 11,325 possible gene-pair SL scores. For validation, 987 gene pairs were selected in the second CDKO experiment. We considered both the cell death pathway and MLEC-iSL prediction probability score in selecting both predicted SL and non-SL gene pairs, which are illustrated in the Results section. We also ranked the SL gene hubs among these 987 pairs and their hub genes.

*Selection of sgRNA in library preparation.* A total of 9,204 sgRNA pairs were synthesized for this study, which correspond with the 987 gene pairs derived from 151 unique genes and 10 sgRNA negative controls targeting non-coding genome sequences (**Supplementary Tables 1-2, 6**). Two sgRNAs were selected for each gene. To minimize transcription efficiency bias, each sgRNA was transcribed using both hU6 and mU6 promoters when paired with another gene. The sgRNAs for each gene were selected based on the methodology described by Tang et al., 2022 [11]. These selected sgRNAs underwent further scrutiny to confirm the absence of XbaI and PacI recognition sites. The finalized oligos comprised two flanking sequences designed for annealing to the vector as well as a central fragment to be arranged as follows: sgRNA 1, ‘TT’+ XbaI cutting site, 20-bp interspacer, PacI cutting site, and sgRNA 2 (**Supplementary Figure 3**).

*Library construction.* The oligo pools were synthesized by Integrated DNA Technologies, Incorporated (IDT; Coralville, Iowa). It was amplified using primer HuU6endF and Scaffold 2R (**Supplementary Table 7**), then ligated to lentiGuide Puro (Addgene #52963) using the GeneArt™ Gibson Assembly HiFi Master Mix (Thermo Fisher Scientific, Waltham, Massachusetts). In the subsequent step, the first step product is digested using XbaI and PacI enzymes (New England Biolabs, Incorporated [NEB], Ipswich, Massachusetts). Simultaneously, a gblock gene fragment (**Supplementary Table 8**) is PCR-amplified using primer XbaI-Etd-Scaffold 1F and PacI-Etd-mU6R, then digested with XbaI and PacI. The ligation of the vector and the gblock fragment is achieved through T4 DNA ligase (NEB). Because the gblock fragment comprises a scaffold (referred to as Scaffold 1) and an mU6 promoter, the finalized plasmid incorporates two sets of promoters, sgRNA and scaffolds, named as hU6-sgRNA1-scaffold1 and mU6-sgRNA2-scaffold2 (**Supplementary Figure 3**).

*Library optimization.* These selected sgRNAs underwent further scrutiny to confirm the absence of XbaI and PacI recognition sites. Additionally, because XbaI digestive activity is blocked by overlapping dam methylation, to prevent potential methylation-related issues, we introduced two additional bases 'TT' immediately preceding the XbaI recognition sequence 'TCTAGA.' The finalized oligos comprise two flanking sequences designed for annealing to the vector as well as a central fragment arranged as: sgRNA 1, ‘TT’+ XbaI cutting site, 20-bp interspacer, PacI cutting site, and sgRNA 2 (**Supplementary Figure 3**). Interspacer length is most important for the success of the double enzyme digestion process.

To optimize cost-effectiveness without compromising efficiency, we conducted cloning efficiency tests using interspacer lengths ranging from three to 20 bp and found 20 bp to be the optimum length to achieve cloning efficiency exceeding 95%. A shorter length could result in reduced cloning efficiency and/or failure of growth in the LB broth media (**Supplementary Table 9**).

In contrast to our laboratory's previously published protocol by Tang et al., 2022, in which 500 nM DNA consisting of 50 oligos was employed as the template in a 50-μl PCR reaction, for this study, we utilized only 10 ng of template DNA to accommodate the substantial size of our oligo pool, comprising a total of 9,204 individual oligos. Using the same amount of oligos as a template in this case would have resulted in an excessively high DNA concentration, potentially leading to non-specific binding of the oligos.

*Cell culture and CRISPR screening.* The 22Rv1 cell line transfected with SpCas9 was a gift from the Xiaoqi Liu lab at the University of Kentucky. The cells were grown in RPMI 1640 media supplemented with 10% FBS, GlutaMAX™, and Penicillin-Streptomycin (Thermo Fisher Scientific). The plasmid library was transfected to HEK 293T with psPAX2 and pMD2.G plasmids (∼1:1:1 molar ratio) to generate the lentivirus supernatant. The MOI was determined following the protocol of Tang et al., 2022. In the CRISPR screening experiment, ten million 22Rv1/Cas9 cells were seeded in each 150mm cell culture plate, and MOI at transfection was controlled within the range of 0.1-0.3. After transfection, the cells were selected by 2ug/mL puromycin (Sigma) for three days. At the end of puromycin selection, the samples were pooled and split into two aliquots. One aliquot was referred to as T0, and the other aliquot was harvested after 10 days as Tend samples. Each sample consisted of about 20 million cells. The experiment was repeated in replicate.

*DNA extraction and preparation of sequencing library.* The genomic DNA was extracted from each sample using GeneJET Genomic DNA Purification Kit (Thermo Fisher Scientific) and subjected to two-round PCR as described in Tang et al., 2022. Six PCR products with different i5 and i7 indices were pooled in equal molar ratios and sequenced by the Illumina NextSeq 2000 platform with PE 100bp kit.

*Guide RNA count mapping and quality control evaluation.* Following sequencing, the reads were mapped using mali-dual-crispr-pipeline v0.4.1 software developed by Amanda Birmingham and Roman Sasik at the Center for Computational Biology, University of California, San Diego (Github link: https://github.com/ucsd-ccbb/mali-dual-crispr-pipeline, accessed on August 1^st^ 2023). These reads were mapped to those targeted sgRNAs (**Supplementary Table 10**). In quality control analysis, we reported the mapping ratio, i.e., the fraction of reads mappable to the targeted sgRNA sequences, Gini index, and correlations among samples (**Supplementary Figure 4, Supplementary Table 11**). The mapped counts were then analyzed using SLKB Python package, and the synthetic lethality scores for each gene pair were calculated (**Supplementary Table 4**).

# **REFERENCES**

1. Long Y, Wu M, Liu Y, et al. Graph contextualized attention network for predicting synthetic lethality in human cancers. Bioinformatics 2021; 37:2432–2440

2. Wang S, Xu F, Li Y, et al. KG4SL: Knowledge graph neural network for synthetic lethality prediction in human cancers. Bioinformatics 2021; 37:I418–I425

3. Liu X, Yu J, Tao S, et al. PiLSL : pairwise interaction learning-based graph neural network for synthetic lethality prediction in human cancers. 2022; 106–112

4. Fan K, Tang S, Gökbağ B, et al. Multi-view graph convolutional network for cancer cell-specific synthetic lethality prediction. Front Genet 2023; 13:

5. Kipf TN, Welling M. Semi-Supervised Classification with Graph Convolutional Networks. 2016; 1–14

6. Vaswani A, Shazeer N, Parmar N, et al. Attention is all you need. Adv Neural Inf Process Syst 2017; 30:

7. Tang S, Wu X, Liu J, et al. Generation of dual-grna library for combinatorial crispr screening of synthetic lethal gene pairs. STAR Protoc 2022; 3:

8. Gökbağ B, Tang S, Fan K, et al. SLKB: synthetic lethality knowledge base. Nucleic Acids Res 2024; 52:D1418–D1428

9. Kanehisa M, Furumichi M, Sato Y, et al. KEGG for taxonomy-based analysis of pathways and genomes. Nucleic Acids Res 2022;

10. Gadepalli VS, Kim H, Liu Y, et al. XDeathDB: a visualization platform for cell death molecular interactions. Cell Death Dis 2021; 12:

11. Tang S, Wu X, Liu J, et al. Generation of dual-gRNA library for combinatorial CRISPR screening of synthetic lethal gene pairs. STAR Protoc 2022; 3:
