# Supplementary figures and images for: Synthetic lethal connectivity and graph transformer improve synthetic lethality prediction
Source: Brief Bioinform. 2024 Aug 30;25(5):bbae425. doi: 10.1093/bib/bbae425 (PMC11361842; doi:10.1093/bib/bbae425)

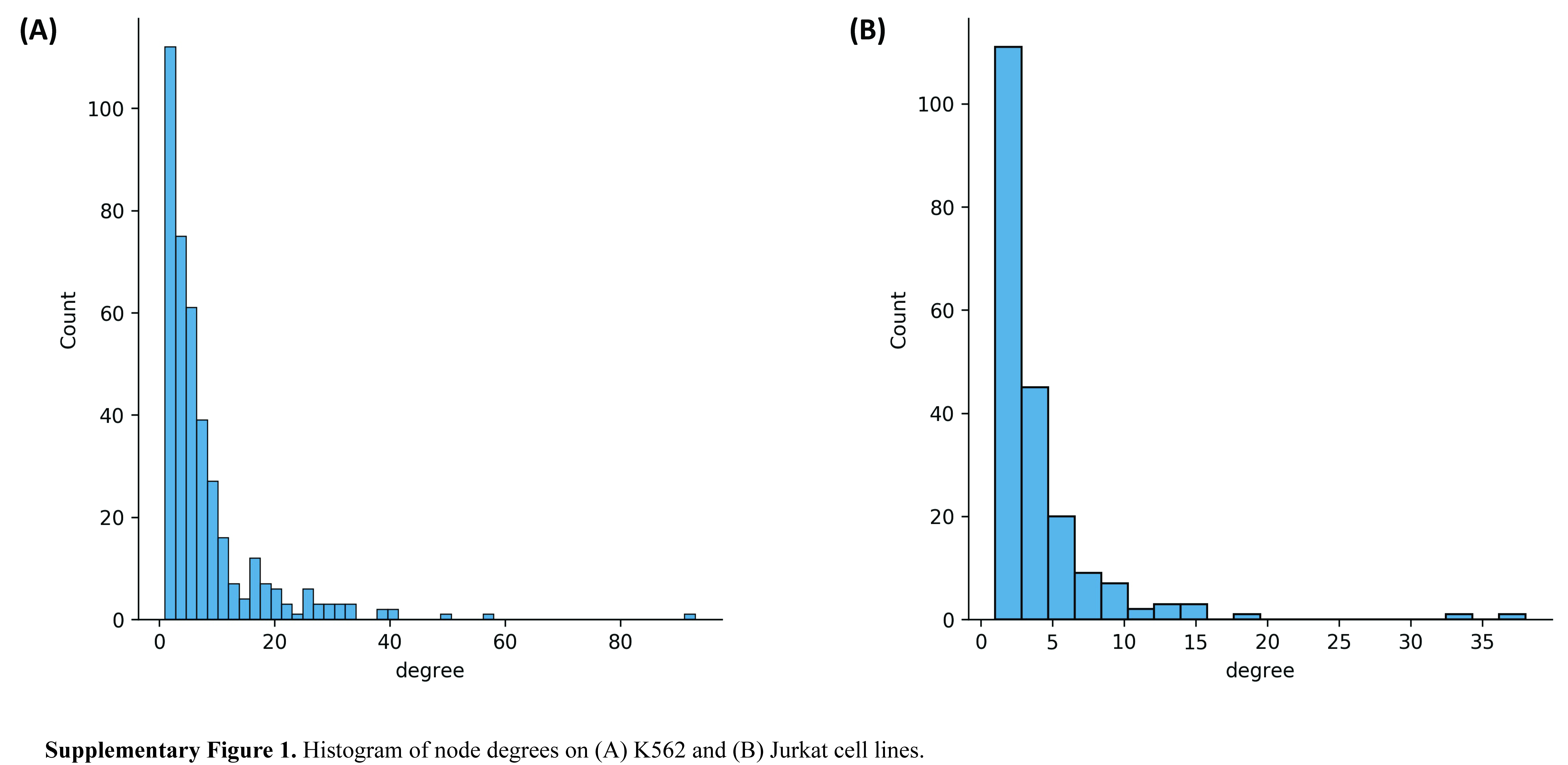

Supplement: Supplemental_Data_bbae425 [file supplemental_data_bbae425.zip › Supplemental_Figure_1 updated.tif]

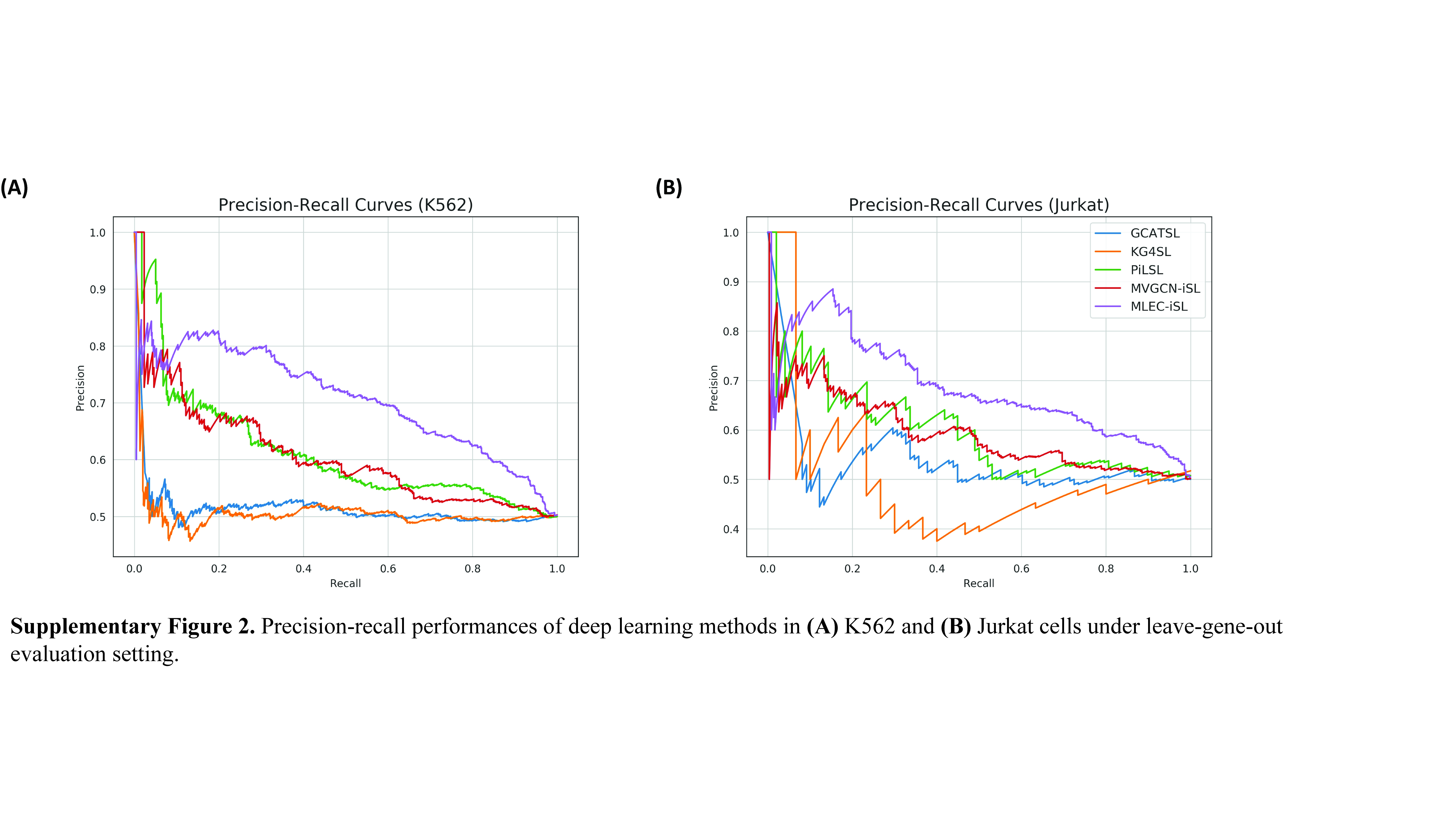

Supplement: Supplemental_Data_bbae425 [file supplemental_data_bbae425.zip › Supplemental_Figure_2 updated.tif]

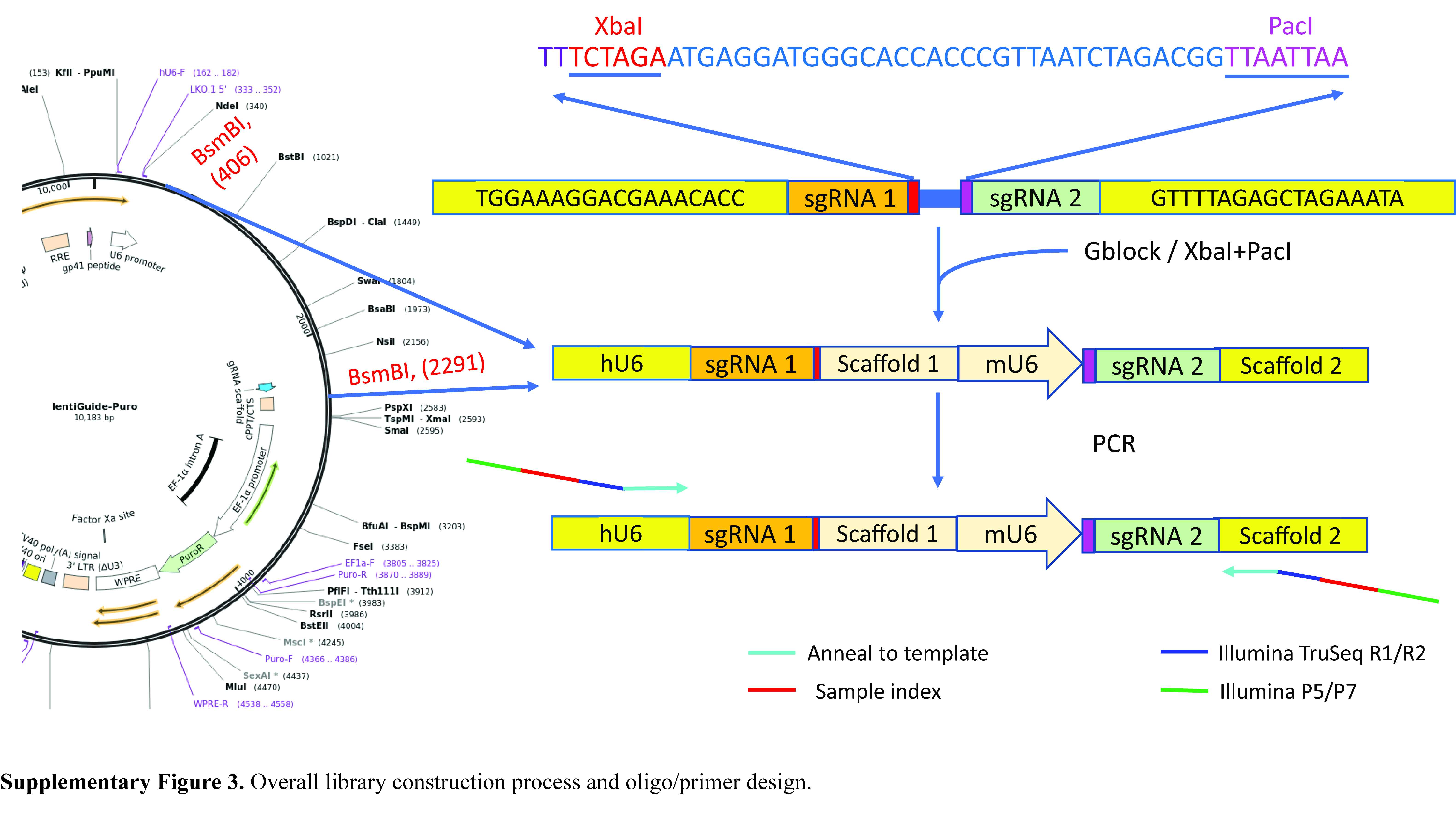

Supplement: Supplemental_Data_bbae425 [file supplemental_data_bbae425.zip › Supplemental_Figure_3 updated.tif]

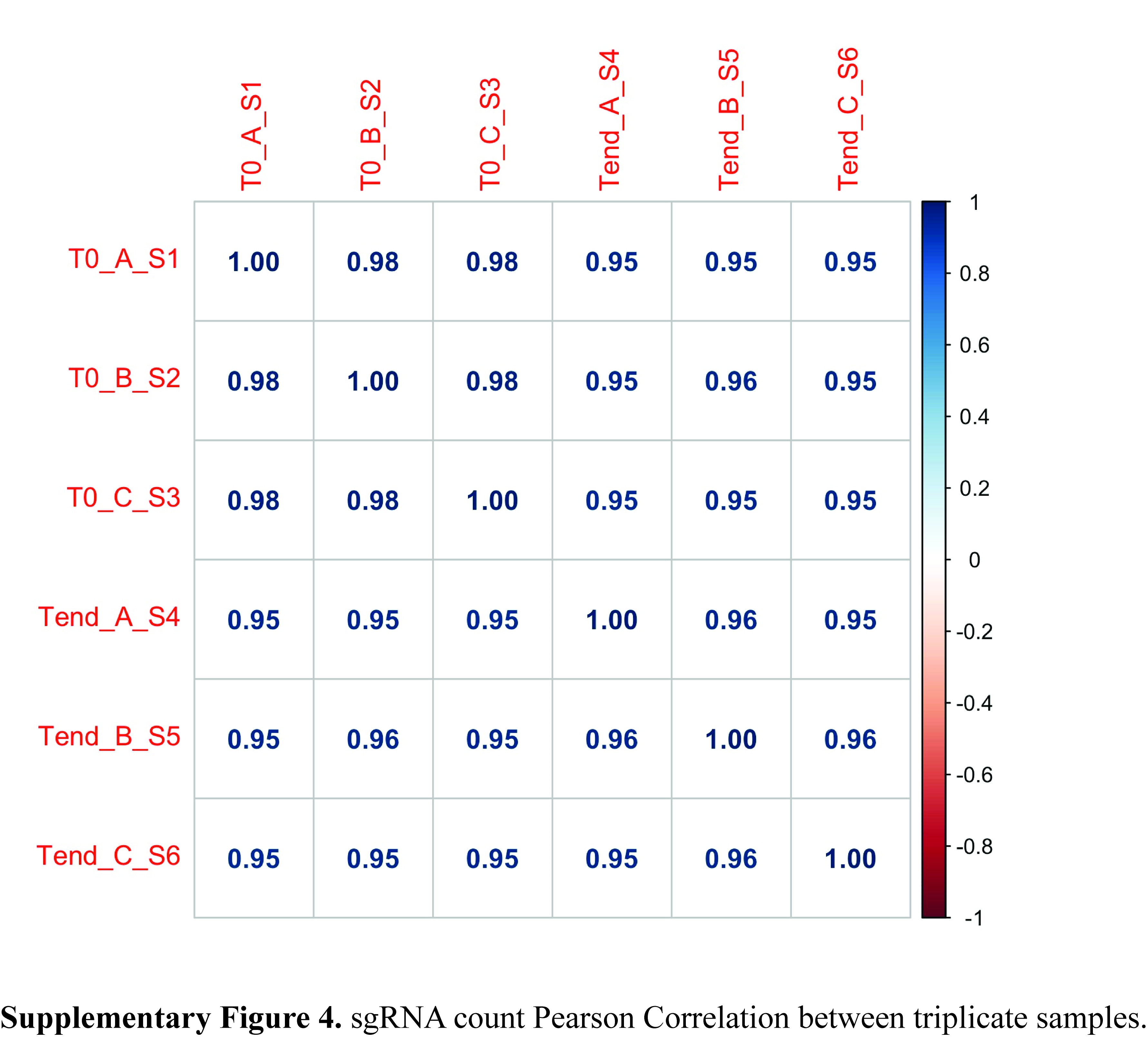

Supplement: Supplemental_Data_bbae425 [file supplemental_data_bbae425.zip › Supplemental_Figure_4 updated.tif]
